# Hypoxic Regulation of Neutrophils in Cancer

**DOI:** 10.3390/ijms20174189

**Published:** 2019-08-27

**Authors:** Daniel Triner, Yatrik M. Shah

**Affiliations:** 1 Department of Molecular & Integrative Physiology, University of Michigan Medical School, Ann Arbor, MI 48109, USA; 2 Division of Gastroenterology, Department of Internal Medicine, University of Michigan Medical School, Ann Arbor, MI 48109, USA; 3 Rogel Cancer Center, University of Michigan Medical School, Ann Arbor, MI 48109, USA

**Keywords:** neutrophil, hypoxia, HIF, cancer

## Abstract

Neutrophils have been well-characterized for their role in the host anti-microbial response. However, it is now appreciated that neutrophils have a critical role in tumorigenesis and tumor progression in the majority of solid tumors. Recent studies have indicated a critical role for hypoxia in regulating neutrophil function in tumors. Furthermore, neutrophil-specific expression of hypoxia-inducible transcription factors may represent a novel therapeutic target for human cancer. In this review, we highlight the function of neutrophils in cancer and the role of the neutrophil hypoxic response in regulating the neoplastic progression of cancer.

## 1. Introduction

**Figure 1:**
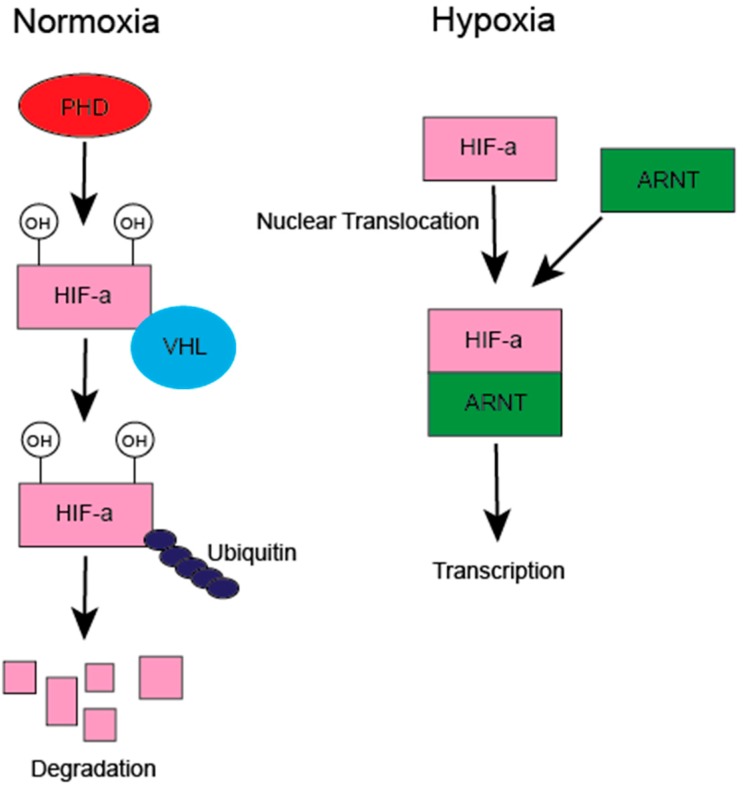
Under normal oxygen concentrations, hypoxia-inducible transcription factors (HIFs) are hydroxylated on two specific proline residues by prolyl hydroxylase domain (PHD) enzymes (HIF1α P402/P564; HIF2α P405/P531), leading to their association with von Hippel-Lindau (VHL), ubiquitination, and proteasomal degradation. In tumors, as oxygen concentrations become limiting, the lack of molecular oxygen leads to HIF stabilization, dimerization with alpha subunit and a beta subunit (ARNT), nuclear translocation, and target gene transcription. In neutrophils, HIFs can also be stabilized in normoxic conditions by inflammatory microenvironments and inflammatory factors, such as bacterial lipopolysaccharide.

The past several decades has determined the immune system plays an integral role in the pathogenesis and progression of human cancers. This has led to the establishment of “tumor-associated inflammation” as an emerging cancer hallmark, and facilitated the development of a new arsenal of the most promising treatments for cancers developed in decades [1,2]. Hypoxia is a characteristic feature of virtually all solid tumors and directly modulates the tumor immune microenvironment [3]. Tumor hypoxia develops as the result of increased oxygen metabolism via uncontrolled cellular proliferation and growth beyond the confines of the existing vasculature. The major signaling pathway downstream of decreased O_2_ concentration is mediated by hypoxia-inducible transcription factors (HIFs). Three HIF isoforms have been described (HIF-1α, HIF-2α, and HIF-3α), which mediate the hypoxic response with overlapping, and occasionally opposing, functions. HIFs consist of a heterodimer of an alpha subunit and a beta subunit (ARNT). Under normal oxygen conditions, HIF α subunits are hydroxylated by prolyl hydroxylase domain (PHD), containing enzymes on two proline residues, which reside in a N-terminal O_2_-dependent degradation domain. This hydroxylation event leads to von Hippel-Lindau-dependent ubiquitination, followed by proteasomal degradation. As oxygen concentrations locally decline in inflammation or in tumors, HIF hydroxylation is inhibited, and HIFs are stabilized. HIFs become transcriptionally active after nuclear translocation, interacting with ARNT, and binding specific HIF target sequences (hypoxia response elements), that are found extensively throughout the genome (Figure 1). HIF expression and stabilization is also directly driven by inflammatory microenvironments and inflammatory factors, such as lipopolysaccharide and cytokines, which we and others have previously reviewed [3,4]. 

In tumors, HIFs have largely been shown to facilitate tumor growth and progression, through direct stimulation of proliferation, angiogenesis, evasion of apoptosis, and invasion [5]. HIFs also regulate metabolic reprogramming of cancer cells [5]. More recently it has been shown that HIFs are key mediators of anti-tumor immune evasion [6]. In addition to tumor-cell intrinsic effects, HIFs can also modulate recruitment and local zonation of immune cells in the tumor microenvironment [7]. Interestingly, local tumor hypoxia also drives immune cell HIF stabilization and transcriptional activation, which phenotypically alters immune cell function and polarization, in order to induce pro-tumorigenic responses in macrophages, T-cells, and B-cells [3]. 

Neutrophils are granulocytic myeloid cells, that are the most abundant circulating immune cell type. Neutrophils serve as first responders to sites of infection and eradicate bacterial infections through local oxygen metabolism, release of cytotoxic granule proteins, and bacterial phagocytosis. Recent attention has been given to the role of neutrophils in the pathogenesis and progression of cancer. Neutrophils, infiltrating into the tumor microenvironment, are termed tumor-associated neutrophils (TANs), and an appreciation for the pathogenic role of TANs in tumorigenesis and tumor progression has now been established [8]. Interestingly, neutrophils express both HIF-1α and HIF-2α and the impact of hypoxia and HIFs on TANs is drawing significant interest. In this review, we will specifically focus on the role of hypoxia and HIFs on TAN function in the progression of cancer, as well as implications for therapy.

## 2. Tumor-Associated Neutrophils (TANs)

Neutrophils are highly prevalent in the vast majority of solid tumors. Recent studies have found that cancer drives neutrophil heterogeneity, and several populations of circulating neutrophils can be detected in cancer. Of these subtypes, neutrophils with pro-tumor and immunosuppressive phenotypes can be isolated, as well as those which maintain mature neutrophil functions [9]. This suggests that cancer-derived factors drive neutrophil plasticity, which can potentiate tumor progression. A meta-analysis of human cancers correlated expression signatures of more than twenty different immune cell types with adverse or favorable outcomes across twenty-five different tumor types. This analysis found that neutrophils had the highest correlation with adverse outcomes [10]. This is in accordance with other studies suggesting, that a high presence of TANs is an independent negative risk prognosticator. Furthermore, studies examining the proportion or neutrophils relative to lymphocytes have established that an increased neutrophil to lymphocyte ratio (NLR) is also predictive of adverse cancer outcomes, and can be used to stratify risk [11]. It is notable that some studies have suggested neutrophils are a positive prognostic indicator in certain cancers at certain stages [12]. The discrepancies in the prognostic ability of neutrophils can, in part, be explained by discrepancies in neutrophil surface makers used for identification. 

**Figure 2:**
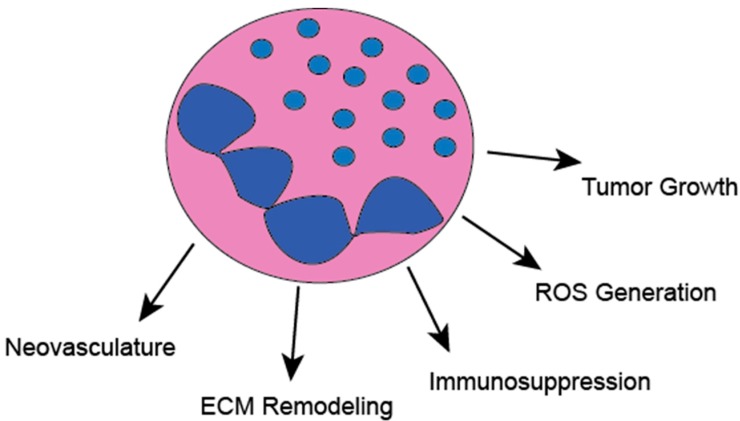
Neutrophils have diverse function in the progression of tumors. Neutrophils directly stimulate tumor angiogenesis. Neutrophils can directly inhibit anti-tumor immunity through the expression of checkpoint molecule PD-L1, stimulate tumor growth through the secretion of neutrophil elastase, facilitate tumor invasion and metastasis through ECM remodeling and generation of neutrophil extracellular traps, and directly contribute to tumorigenesis through the generation of genotoxic reactive oxygen species.

The role of TANs in cancer has been controversial, however, the vast majority of studies suggest neutrophils can affect all stages of tumorigenesis; including initiation, propagation, invasion, and metastasis (Figure 2). For example, studies using mice with deletion of CXCR2, which prevents neutrophil and immune suppressive myeloid cell infiltration into tumors, have shown decreased colon tumorigenesis [13]. A landmark study showed that neutrophils can promote the metastatic spread of breast cancer through the inhibition of CD8^+^ T-cells [14]. Neutrophil extracellular traps (NETs), normally known for trapping invading bacteria, can facilitate the metastasis of circulating tumor cells [15]. Several other intricate mechanisms for pro-tumorigenic neutrophil functions have been identified comprising direct stimulation of tumor cell growth, antitumor immune suppression, and stimulation of neo-vasculature [8]. Additionally, it was shown in hepatocellular cancer models that neutrophils can regulate tumor stem cell dynamics through secretion of BMP2 and TGF-β2 [16]. Furthermore, neutrophils also have the capacity to alter the tumor immune microenvironment, and can increase tumor growth, through the recruitment of regulatory T-cells [17]. 

It is now appreciated that neutrophils may have anti-tumorigenic function as well. Neutrophils, expressing the hepatocyte growth factor receptor c-MET, inhibit growth of several cancer types, through the release of tumor necrosis factor α (TNFα) and nitric oxide (NO) [18]. We have recently shown that genetic neutrophil depletion enhanced the growth and invasion of inflammation-driven and sporadic colon tumors in mice, through the restriction of tumor associated bacteria [19]. Neutrophil ablation led to a high IL-17-dependent B-cell influx [19]. Neutrophils were shown to directly stimulate anti-tumor immunity in early stage human lung cancer [20]. Interestingly, rare sub-populations of TANs antigen presenting cells and stimulate anti-tumor T-cells, through the presentation of tumor-associated antigens in human lung cancer [21].

Many of the observed discrepancies among the role of TANs in cancer are likely reflective of temporal dynamics and neutrophil heterogeneity. Temporally, it has been suggested that the earliest neutrophils in cancer are anti-tumorigenic. As tumors rapidly evolve, secreted factors drive neutrophil plasticity towards pro-tumorigenic subsets and decrease their cytotoxic capacity [22]. The tumor-derived factors regulating neutrophil evolution have not been explored but may be related to tumor hypoxia. Several subsets of neutrophils with varying functions have been identified in mice and humans with cancer. A landmark study showed that, neutrophils are highly plastic and can transition between phenotypically divergent anti-tumorigenic and pro-tumorigenic subsets, termed N1 and N2 [23]. This is analogous to the polarization of macrophages to M1 or M2 and T-cell polarization. In neutrophils, this transition is thought to be regulated by a transforming growth factor β (TGFβ) and interferon γ (IFNγ). In macrophages, tumor hypoxia promotes a phenotypic shift towards the pro-tumorigenic M2 macrophage through tumor-derived lactic acid generation [24]. The role for hypoxia and HIFs in the plasticity of TANs has yet to be addressed. 

## 3. HIF Regulation of the Tumor Immune Microenvironment

The tumor microenvironment is a complex assortment of tumor cells, endothelial cells, stromal cells, and immune cells. Many of the infiltrating immune cells, such as macrophages, neutrophils, myeloid-derived suppressor cells, and T-cells facilitate tumor growth, progression, and evasion of the immune system. Several of these sub-populations of pro-tumor immune cells are recruited through hypoxia-dependent factors. For example, immune suppressing regulatory T-cells (Tregs) are recruited to ovarian tumors by hypoxia-dependent expression of CCL28 [25]. In breast cancer, the hypoxic secretion of oncostatin M promotes recruitment of macrophages [26]. Once these cells are recruited into hypoxic tumor microenvironments, immune cell HIF activation can regulate downstream effector functions, such as pro-tumorigenic polarization. For example, macrophage deletion of HIF-2α was shown to reduce macrophage-dependent cytokine production and tumor burden in murine models of liver and colon cancer [27]. The role for hypoxia and HIFs on the function of neutrophils has been less well characterized than that of other immune cell types, but current literature suggests a key role in the pathogenesis and progression of cancer.

### 3.1. Hypoxic Regulation of Neutrophil Inflammatory Responses

Tumor hypoxia extends to infiltrating immune cells, and it is evident that many immune cells, TAMs in particular, selectively localize to hypoxic regions of tumors, which drives spatial variation in function [28]. Moreover, the infiltration of immune cells, neutrophils in particular, contributes to local hypoxia, through respiratory burst-dependent oxygen metabolism [29]. The role for neutrophil-specific HIF expression in cancer has not been well characterized. However, several studies have examined neutrophil-specific HIF functions in inflammation and infection. Neutrophils express both HIF-1α and HIF-2α. The activation of these transcription factors in neutrophils is induced by hypoxia, but is also directly induced by incubation with heat-killed bacteria and bacterial lipopolysaccharides, showing that inflammation- and hypoxia-signaling are closely interconnected. Furthermore, circulating neutrophils, isolated from patients with chronic inflammatory disorder, show robust HIF activation. 

Hypoxia augments neutrophil inflammation and enhances degranulation [30]. The deletion of VHL, which stabilizes HIFs in physiologic oxygen concentrations, robustly increased neutrophil inflammatory responses. Moreover, the deletion of HIF-1α impaired neutrophil ATP generation, bactericidal activity, and motility [31]. This was further corroborated in a study showing reduced bactericidal activity in neutrophils lacking HIF-1α [32]. Neutrophil HIF-1α is also essential for anti-fungal activity [33]. Pharmacologic HIF-1α stabilization can augment the anti-microbial effects of neutrophils. Less is known about neutrophil-specific HIF-2α. In zebrafish, it was shown that HIF-2α gain of function mutation prolonged the inflammatory response [34]. This same study showed the myeloid-specific deletion of HIF-2α resulted in neutrophilic inflammation in a murine lung injury model [34]. Interestingly, studies from human patients with heterozygous *VHL* mutations have shown increased neutrophil resistance to apoptosis and augmented phagocytic capacity [35]. Collectively, these studies suggest a bi-directional crosstalk, whereby inflammation directly promotes HIF expression in neutrophils, and this leads to an increase in neutrophil inflammation. Chronic inflammation is a cancer risk factor and neutrophils have been described as a critical link between chronic inflammation and cancer [36].

Major isoforms of PHDs that regulate HIF hydroxylation and degradation have been described and are all expressed in neutrophils (PHD1, PHD2, and PHD3). The deletion of PHD2 or small molecule PHD inhibition dramatically augmented the neutrophil inflammatory response, motility, and survival in *Streptococcus pneumonia* infection [37]. This effect was dependent on increased glycolytic flux and glycogen storage suggesting that HIF integrates metabolism with inflammation and survival [37]. Interestingly, the deletion of PHD3 dramatically reversed hypoxia-induced neutrophil survival [38]. In this model it was proposed that PHD3 mRNA was upregulated by hypoxia downstream of HIF-1α and regulated expression of pro-survival molecules [38].

### 3.2. Hypoxic Regulation of TAN Mobilization

Studies of human and mice have shown a relative increase in the number of circulating blood neutrophils in cancer. Tumor hypoxia is a critical mediator of pro-tumor immune cell recruitment through the secretion of cytokines and chemokines. HIF-dependent mechanisms of TAM and T-reg recruitment are well-characterized. TAN recruitment and influx is critical for cancer initiation and progression and tumor hypoxia, through HIF-dependent secretion of cytokines is a major regulator of this process [39]. Indeed, one of the earliest studies linking hypoxia and neutrophil recruitment, showed that hypoxic human intestinal epithelial cells promote neutrophil migration, through the induction of IL-8 [40]. Hypoxia-induced IL-8 is also a key signaling axis in neutrophil recruitment in cancer [41]. The infiltration of neutrophils in tumors or the sites of inflammation requires endothelial cell binding and extravasation, and it has been shown that culturing endothelial cells in hypoxia increases neutrophil binding, through the upregulation of platelet-activating factor [42]. HIF-1α also stimulates the expression of the cell surface adhesion molecule, β2 integrin, on neutrophils [43]. 

Similar results have been derived from spontaneous murine tumor models. A study in a mouse model of uterine cancer found that neutrophils largely resided within hypoxic foci, and the culture of uterine cancer cell lines in hypoxia led to the expression of potent neutrophil chemo-attractants; CXCL1, CXCL2, and CXCL5 [44]. Interestingly, neutrophils restricted tumor growth in this model, by promoting tumor cell shedding from the underlying basement membrane. Recently, we showed that hypoxia, through HIF-2α, mediates the recruitment of neutrophils into inflammation-induced colon tumors, by the direct transcriptional regulation of the neutrophil chemoattractant CXCL1 [39]. CXCL1 functions by binding its cognate receptor, CXCR2, expressed on neutrophils leading to cytoskeletal rearrangement to promote influx into tumors or sites of inflammation. We showed that the inhibition of this pathway with peptide inhibitors was sufficient to reduce inflammation-induced colon tumors in mice. This is in line with other studies showing that the ablation or inhibition of CXCR2 is sufficient to decrease colon tumorigenesis in mice [45]. 

It has been postulated that cancer metastasis to distant sites requires the formation of a premetastatic niche, mediated by immune cells, to generate ideal environmental conditions for cancer seeding [46]. Tumor hypoxia is a critical regulator of myeloid cell and neutrophil infiltration to the premetastatic niche. The role for neutrophils in this process is controversial. In breast cancer models, hypoxia induced lysyl oxidase, or Kit ligands promoted myeloid cell infiltration and lung cancer metastases [47,48]. Furthermore, tumor hypoxia-derived factors were shown to facilitate the mobilization of immunosuppressive myeloid cells and Natural Killer cells to the lung premetastatic niche [49]. However, others have shown an opposing role for neutrophils in the premetastatic niche, and anti-metastatic, hydrogen peroxide (H_2_O_2_)-producing neutrophils were recruited to premetastatic sites, through the expression of the HIF target gene CCL2 [50]. 

### 3.3. Hypoxic Modulation of TAN Function

The half-life of circulating blood neutrophils is thought to be on the order of 7-h. Interestingly circulating neutrophil lifespan is dramatically increased in cancer patients through an unknown mechanism, but it is likely to be the downstream of tumor secreted factors [8]. It is also believed that TAN have increased lifespan in cancer patients. Hypoxia is sufficient to decrease neutrophil apoptosis. In murine lung injury models, the deletion of HIF-2α, using the myeloid-specific *LysM*-Cre, increased neutrophil susceptibility to apoptosis. On the other hand, HIF-2α gain-of-function mutations decreased neutrophil sensitivity to apoptosis in human neutrophils. There is also an important role for HIF-1α in the regulation of the neutrophil lifespan. Human and murine neutrophils have decreased apoptosis when cultured in hypoxia via a HIF-1α/NFkB signaling axis [51]. This data is consistent with zebrafish models where HIF-1α was also critical in decreasing neutrophil apoptosis [52]. These studies highlight an essential role of HIFs in the longevity of neutrophils, and suggest an analogous mechanism exists in hypoxic tumors, although this has not yet been studied. 

**Figure 3:**
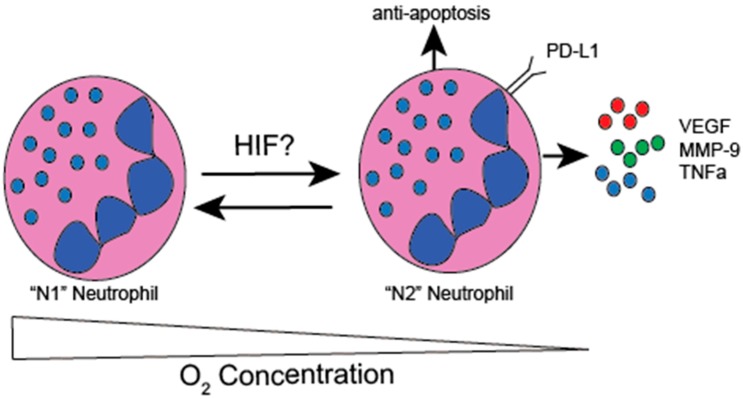
HIF expression regulates diverse neutrophil functions. HIFs promote neutrophil longevity and evasion of apoptosis. HIF regulates key neutrophil inflammatory responses, including NF-kB activation and bactericidal capacity. HIF regulates neutrophil expression of pro-tumor factors TNFα, VEGF, and MMP-9. Furthermore, HIF can directly induce expression of anti-tumor T-cell molecules, such as PD-L1. Currently it is unclear the functional role for hypoxia and HIFs in regulating neutrophil plasticity and their transition from anti-tumor N1 to pro-tumor N2.

There is an emerging role for neutrophils as a critical suppressor of the anti-tumor immune response. In gastric cancer, neutrophils suppress antitumor T-cells through the expression of the cell surface protein programmed death ligand 1 (PD-L1) [53]. Recent studies show that PD-L1 is a direct HIF transcriptional target in immune suppressive myeloid cells [54]. Neutrophil-derived arginase represses anti-tumor immunity, through the local metabolism of arginine to L-ornithine, and is a known target of HIF-2α [55]. Collectively, these studies suggest that hypoxia contributes to neutrophil plasticity and differentiates neutrophils away from an anti-tumor phenotype to an immune suppressive phenotype to foster tumor growth (Figure 3). 

We previously identified the pro-inflammatory cytokine, TNFα, as a direct transcriptional target of HIF-2α, which has been critical for heightened inflammation in experimental colitis [56]. TNFα has been identified as a critical regulator of cancer cell growth in murine colitis-induced colon cancer and can promote recruitment of immune suppressive immune cells [57,58]. Neutrophils are a major source of TNFα, and it is likely that neutrophil-derived TNFα, downstream of HIF-2α activation plays an important role in inflammation-induced colon tumor growth [59] (Figure 3). 

Neutrophil secreted factors impact tumor growth. Neutrophil elastase (NE) directly stimulates the proliferation of lung cancer growth, through the degradation of insulin receptor substrate-1 (IRS-1) and can be directly induced by hypoxia [32,60]. Several studies suggest a key role for neutrophils in the generation of neo-vasculature. Neutrophils are a major source of vascular endothelial growth factor (VEGF) in tumors, and VEGF is a well-characterized direct HIF target gene [61,62]. Moreover, the secretion of matrix metalloproteinase-9 (MMP-9), which promotes angiogenesis through the interactions with VEGF, was shown to be highly upregulated by hypoxia, and was directly targeted by HIF in neutrophils [63,64]. MMP-9 also directly promotes tumor invasion through extracellular matrix degradation (Figure 3).

## 4. Future Perspectives

HIFs were discovered less than 30 years ago, and since that time, the importance of these molecules in normal physiology and the disease state has been characterized. In cancer, the role for hypoxia and HIFs in the neoplastic progression of solid tumors has been well-documented. HIFs regulate diverse cell functions and impact all aspects of tumorigenesis, including the tumor inflammatory response. Cancer cell expression of HIFs drives tumor cell proliferation and shapes the tumor immune microenvironment to inhibit the anti-tumor immune response. Once thought to be undruggable, structural studies have deciphered a ligand-binding pocket at the interface of HIF-2α and ARNT, that has led to the development of potent small-molecule inhibitors that dissociate HIF-2α from ARNT. This ligand binding domain is specific to HIF-2 α but not HIF-1α. Recently, two major studies showed the therapeutic potential for targeting this ligand-binding domain of HIF-2α, with small molecule inhibitors in clear cell renal cell cancer [65,66]. This work has been extended to human clinical trials with high safety and promising early results [67]. The data reviewed herein suggest that the inhibition of HIF-2α may provide anti-cancer activity through the direct inhibition of tumor cell intrinsic HIF-2α, as well as through the inhibition of neutrophil HIF-2α. No specific inhibitors of HIF-1α are currently available. 

The role for neutrophils in the pathogenesis of cancer is complex, and this intricacy is likely related to diverse subpopulations of neutrophils, localized within tumors. In spite of these heterogenous populations, the majority of studies suggest that the inhibition of neutrophils can robustly induce tumor regression. Furthermore, studies of neutrophil expression signatures and NLR suggests that neutrophils are an adverse prognostic indicator in human cancers. Most studies of neutrophil function in cancer have relied upon the use of monoclonal neutrophil depleting antibodies, CXCR2 peptide mimetics, and small molecular inhibitors. Robust anti-tumor responses with these neutrophil inhibitors have been shown in pre-clinical models across several different tumor types [45,68,69]. In fact, clinical trials, specifically targeting CXCR2, are underway in human cancers. Studies have also shown efficacy in the targeting of neutrophil elastase in pre-clinical colon cancer models. It is likely that a further understanding of neutrophil heterogeneity may drive the development of novel therapeutics, which will allow fine-tuning and targeting of pro-tumor neutrophil subsets, while sparing those that are anti-tumor. Advances in single-cell RNA sequencing may provide the opportunities to more precisely define distinct neutrophil subsets in cancer and broaden the current therapeutic arsenal.

Immune cell hypoxia and the expression of HIFs play a pivotal role in their function and longevity in tumors. The role for neutrophil-specific HIFs in cancer has not been well-studied. It has been well-documented that HIFs are essential regulators of neutrophil longevity, inflammation, and bactericidal activity. It is clear that tumor hypoxia is a major contributing factor to neutrophil recruitment to tumors, through the direct HIF transcriptional regulation of neutrophil chemo-attractants. The gaps in our current knowledge of HIF function, in intra-tumoral neutrophils, has been in part due to the lack of tools to study neutrophil-specific gene deletion. Recently, novel tools, such as the *Mrp8*-promoter-driven Cre mouse, have provided the means for neutrophil selective gene deletion, and will allow for the intricate investigation of the role of HIFs in neutrophil function in mice [70]. To date, most studies have relied upon the *LysM*-promoter-driven Cre expressing mice, which target all myeloid cells. This can make the interpretation of studies, deleting HIFs via *LysM*-Cre, challenging to parse out the primary contribution of neutrophils or macrophages on cancer or inflammation. 

Many unanswered questions and areas for interrogation remain. For example, the precise role for HIFs in the polarization of neutrophils from anti-tumor N1 to pro-tumor N2 subtypes is not known. Furthermore, neutrophil-specific HIF target genes have not been parsed out. A recent analysis in tumor-bearing mice compared the gene expression signatures between normal neutrophils, TANs, and immature myeloid cells. Interestingly, this analysis found that TANs had tremendous upregulation of the expression of inflammatory cytokines and chemokines, several of which are known HIF target genes [71]. It has been suggested the HIF-dependent metabolic reprogramming regulates key myeloid cell functions and it would be interesting to determine the role of these metabolic changes in neutrophils in cancer. The future should provide exciting new insights in the biology of HIFs in neutrophils and the intersection of these factors in human cancers.
